# Health and fitness trends in the post-COVID-19 era in the United Arab Emirates: A cross-sectional study

**DOI:** 10.3934/publichealth.2024044

**Published:** 2024-07-05

**Authors:** Alexios Batrakoulis, Željko Banićević, Ivana Banićević, Ashokan Arumugam, Ivan Marović, Nemanja Krstić, Saša Obradović

**Affiliations:** 1 Department of Physical Education and Sport Science, University of Thessaly, Trikala, Greece; 2 Department of Physical Education and Sport Science, Democritus University of Thrace, Komotini, Greece; 3 HERC – Health, Exercise & Research Center, Dubai, United Arab Emirates; 4 Department of Physiotherapy, College of Health Sciences, University of Sharjah, Sharjah, United Arab Emirates; 5 Neuromusculoskeletal Rehabilitation Research Group, RIMHS – Research Institute of Medical and Health Sciences, University of Sharjah, Sharjah, United Arab Emirates; 6 Sustainable Engineering Asset Management Research Group, RISE – Research Institute of Science and Engineering, University of Sharjah, Sharjah, United Arab Emirates; 7 Adjunct Faculty, Manipal College of Health Professions, Manipal Academy of Higher Education, Manipal, Karnataka, India; 8 Sport & Wellness Office, Abu Dhabi University, Abu Dhabi, United Arab Emirates; 9 Student Activities Department, Middlesex University, Dubai, United Arab Emirates

**Keywords:** United Arab Emirates, fitness survey, trends, top programs, top services, ACSM survey

## Abstract

The health and fitness (H&F) sector is rapidly evolving and appears to be a vibrant space for industry stakeholders with a great potential globally. This observational study aimed to identify the most popular trends related to H&F services in the United Arab Emirates (UAE) for the first time, focused on the industry status after the coronavirus (COVID-19) pandemic, and aimed to detect potential differences with the recent results observed in other countries or regions. Additionally, a chi-square analysis was applied to determine the significant differences between trends and demographics, such as sex, age, experience, and work status. A national online survey was conducted, and applied the methodology of similar international surveys that have been carried out by the American College of Sports Medicine since 2006. In particular, simple random sampling was utilized through an online questionnaire sent to 2771 professionals involved in the UAE's H&F sector. In total, 322 responses were collected with a response rate of 11.6%. The 10 most popular H&F trends in the UAE during the post-COVID-19 era were exercise for weight loss, personal training, traditional strength training, employing certified exercise professionals, boxing, kickboxing, mixed martial arts, youth athletic development, high-intensity interval training, massage, bodyweight training, and wearable technologies. Exercise for weight loss (*p* = 0.001) and lifestyle medicine (*p* = 0.032) were more popular among females compared to males, while traditional strength training (*p* = 0.035) was reported more frequently by males. Going to health clubs and spas (*p* = 0.001) and practicing yoga (*p* = 0.011) were more popular trends among middle-aged (36–64 years) respondents compared to young ones (18–34 years). Athletic development (*p* = 0.042) was more frequently reported by non-practitioners (students) compared to practitioners (part- and full-time employees). The present results are partially in line with those reported in other recent national, regional, and global surveys, which investigated the top H&F trends after the COVID-19 pandemic. Importantly, the main outcomes of this study indicate that the industry stakeholders should focus on in-person H&F services since trends related to technology and digital services are not currently popular nationwide. Moreover, the majority of the top trends were more traditional and rooted activities, which showed that the current status of the H&F sector has established particular training services, programs, and products in the UAE.

## 1. Introduction

The health and fitness (H&F) sector is considered a fast-growing business area, demonstrating significant potential for evolution and innovation globally [1]. Importantly, this dynamic space promotes various activities related to physical activity, exercise, and wellness among people of all ages, races, and training experience levels; moreover, it aims to support public health and disseminate the critical role of an active lifestyle in health and well-being after the coronavirus pandemic (COVID-19). Specifically, the H&F sector sees recovery in the post-COVID-19 after almost three challenging years that negatively affected business and consumer habits. However, the key role of regular bodily movement has been extensively highlighted and the positive influence of various fitness services, programs, and products has been extensively observed [2]. In the United Arab Emirates (UAE), the number of fitness facilities and members has been increasing in the past few years, showing a dynamic profile of the national H&F sector compared to developed countries. Interestingly, the UAE's H&F sector appears an emerging and competitive market, demonstrates attractive business and professional development opportunities among industry stakeholders, and aims to support public health through positive and inclusive fitness experiences. Programs that are relevant to Millennials, Generation Z, and women are on the rise nationwide [3]. Interestingly, multipurpose gyms and boutique fitness studios offering services in a luxurious environment seem to be an emerging trend in fitness facilities in the UAE, which is in line with the current status of the H&F sector worldwide concerning fitness settings, with the greatest potential for growth and innovation [4]. During the COVID-19 lockdown, the leading causes of unhealthy behavior in the UAE were increased food intake and decreased physical activity, followed by increased weight, decreased sleep, and increased smoking. Even before the COVID-19 pandemic, the UAE population exhibited a considerably elevated occurrence of sedentary behavior and physical inactivity. It is worth mentioning that one in two individuals are physically inactive, two in three individuals are overweight, and one in three individuals have obesity among adults in the UAE, indicating a similar epidemiological status to that reported in the Western world [5],[6]. On the other hand, the physically active way of living has been underlined as an effective behavior to reduce inactivity and obesity that attract various lifestyle-related chronic diseases worldwide [7],[8].

The UAE is one of the world's wealthiest states, demonstrating one of the highest gross domestic products per capita worldwide [9]. Thus, the local fitness market has shown tremendous growth in the past 10 years, attracting not only residents, but mostly foreign entrepreneurs and workers. Interestingly, the UAE's health, fitness, and wellness sector is a very competitive business arena, especially between international and domestic players. The major growth market drivers are population growth, high-net-worth individuals, increased obesity rates, government initiatives, and an increase in dedicated gyms for women [3]. The prevalence of the most common lifestyle-related health issues is rising at an alarming rate, setting public health under pressure while reducing the quality of life in adults [10]. Noticeably, the large majority of the adult population have been affected by various cardiometabolic health diseases in the UAE [10]. A significant proportion of deaths in the UAE, approximately 77%, are attributed to non-communicable diseases (NCDs), with prevalent risk factors including an elevated body mass index, an increased systolic blood pressure, elevated fasting plasma glucose levels, and elevated total cholesterol levels [10]. Hence, the rising awareness regarding sedentarism and excess weight associated with major health issues seems to be a top priority to strengthen the rationale for fitness services and to support a national public health policy [11]. Noticeably, the UAE's H&F sector is rapidly increasing its size, showing an increased number of facilities, club members, and practitioners involved in this well-established market. In particular, the UAE's physical activity, exercise, and wellness sector displays a total annual revenue of USD 0.36 billion in 2020; however, this is expected to exceed USD 0.6 billion by 2025, showing a great growth potential [1],[11].

A worldwide annual survey that emphasized the top H&F trends was conducted by the American College of Sports Medicine (ACSM) over the past 19 years. Such an observational study aimed to investigate the most popular exercise modes, training settings, and fitness services in the industry at the global level and sought to identify the influence of those trends on practitioners and other industry stakeholders [12]–[29]. Research of this kind may help not only industry stakeholders, but also consumers to consider investing in good practices that promote safe, effective, and pleasant fitness experiences. Importantly, such cross-sectional studies may support gym operators and employees by highlighting specific areas in which they have to pay more attention. Such an approach provides all involved parties with new professional opportunities, focusing on a new landscape linked to evident outcomes [29]. The current status of trends in the UAE's H&F sector has not been investigated nationwide. Taking this into consideration, it is evident that the lack of an in-depth analysis of the most popular H&F trends augments the gap between science and practice at the national level. In contrast, a wide-ranging examination has been previously conducted by the ACSM, focused on several countries and regions, collected data, and published comparative reports at the international level [30]–[34]. Noteworthy, several national and regional observational studies of this kind have replicated the ACSM's methodology, distributed applicable results, and aimed to raise awareness of the most attractive H&F trends at the international level [35]–[44].

This cross-sectional study is the first-ever survey that collects data from the UAE and compared the findings with those reported for various countries and regions [29]. From Asia, only data from China, Iran, and Turkey have been already published in the past [41],[43],[44]. Taking this into account, such a national survey will yield insights into the H&F trends from another Asian country, and this is important given that the UAE has been identified as an emerging fitness market, attracting suppliers, fitness club chains, operators, educators, and exercise professionals from all over the world. Hence, the objectives of the present study are as follows: a) to identify the most popular H&F trends in the UAE in the post-COVID-19 era; and b) to compare these trends between various countries and regions. In summary, this observational study may benefit H&F sector stakeholders, given that they will be capable of bringing their services, programs, and products into line with the current top trends in the post-COVID-19 era at the national level. Moreover, leading stakeholders and policy-makers can use the present report results to make up-to-date decisions, thus supporting public health through greater customer retention, engagement, and loyalty.

## 2. Materials and methods

### 2.1. Study design

A cross-sectional study of H&F trends was carried out, and utilized an online survey and a descriptive approach. The present observational study used the same methodology with relevant surveys conducted by the ACSM. In particular, this study applied similar criteria to those that have been widely included in relevant national [35]–[44], regional [30]–[34], and worldwide [12]–[29] surveys of H&F trends since 2006. In the present study, data were collected through a national survey to report the main results at both the local and international levels. That being said, a comparison between the present outcomes and those recently reported in other countries and regions was conducted [29]. In short, the web-based survey was developed to identify the H&F trends (not fads) that were considered popular because of their impact on the industry, while demonstrating an increased attractiveness among industry stakeholders in the UAE. Therefore, a distinction between a “fad” and a “trend” following the dictionary was included in the introduction of the online survey, which aimed to help the respondents recognize the difference between these two key terms as previously articulated [12]–[29].

### 2.2. Sample recruitment and inclusion criteria

Only adult participants with any involvement in the UAE's H&F sector were eligible to participate in the study. Participants were mainly recruited through databases of contacts of local institutions and organizations. In particular, the survey was supported and distributed as follows: HERC- Health, Exercise & Research Center (28%), DSC- Dubai Sports Council (22.7%), Abu Dhabi University (20.8%), University of Sharjah (17.4%), and Middlesex University Dubai (11.1%). The web-based survey was sent electronically to 2771 individual contacts in total. All contacts were people highly involved in the national H&F sector, exhibiting various occupations, working experiences, and education levels. Additionally, numerous posts on social media and relevant websites were made to promote the online survey and increase the awareness of this research project nationwide.

### 2.3. Data collection tool

A valid and reliable methodology that was widely used in similar research attempts over the past two decades by the ACSM was applied. In particular, a group of technical experts with a broad experience in the H&F space as practitioners, educators, and/or researchers was recruited to identify a list of fitness trends [29]. This group (*n* =30) of industry experts and academics from all over the world was involved in a pilot study to complete the list of candidate H&F trends from which the online survey was developed after verifying the consistency of the questionnaire and modifications were applied accordingly [40]. The internal consistency of the questionnaire was assessed by calculating the alpha–Cronbach's coefficients, where *α* = 0.74 indicated a respectable level of internal consistency among the items of the questionnaire. Hence, an electronic questionnaire using an online survey platform (Google Forms) was developed, including 46 related trends that were retrieved from several sources and the experts' personal experiences. The original ACSM survey was adapted to local needs by making minor amendments. These modifications included the inclusion of recovery-based therapies, such as water immersion and massages, which have gained popularity in the UAE after the COVID-19 pandemic. Additionally, changes were made to the demographics section, replacing the question about country alongside the nationality, and inquiring about the city where participants currently work rather than their place of residence. Furthermore, six certifications were added, as well as the occupation of a strength & conditioning coach as a primary profession. The “where do you work” question now provided options for various locations/freelancers. To capture a wider range of respondents, those who were interested in fitness and health but were not exercise professionals were given the option to select “I am not an exercise professional” for the current work status question. Finally, the participants were asked if they were members of specific organizations, namely Register of Exercise Professionals (REPs) UAE and/or UAE Body Building & Fitness Federation, both of which are currently the only organizations offering memberships in the UAE. In the questionnaire, a brief description was provided for each trend, which supported respondents to efficiently clarify each option as previously reported [30]. The potential trends were assessed using a 10-point Likert scale ranging from 1 (least likely to be a trend) to 10 (most likely to be a trend), as previously described [30]. The questionnaire required no more than 12–15 minutes to be completed. In the questionnaire, various demographic questions were included regarding gender, age, region, education, certification(s), occupation, experience, work status, and work setting, as previously reported [30]. The questionnaire was provided in English with minor region-based amendments (stated above) from the original version designed by the ACSM. The region-based questionnaire was approved by the ACSM's Work Group, which was in charge of overseeing all international surveys.

### 2.4. Recruitment and study period

The survey was carried out electronically from July 5, 2023, to August 13, 2023 (5.5 weeks), and re-opened from September 4, 2023, to October 15, 2023 (6 weeks), for a total of 11.5 weeks of data collection. No financial or material incentives were offered to attract respondents to the survey. The respondents could choose to stay anonymous or leave their name and contact details and enter a prize draw, where they had a chance to win one of five 500 AED vouchers that could be used for fitness services at HERC. Additionally, they received a certificate of participation for taking part in the survey. Two email reminders were sent to all database contacts during the study period. All participants signed a digital informed consent letter before submitting their answers as part of the electronic survey. All mandatory specifics concerning the research aims, the confidentiality of the information, and the right to withdraw participation in the study were included on the first page of the electronic survey.

### 2.5. Data analysis

This cross-sectional survey was methodologically grounded in numerous similar observational studies broadly conducted by the ACSM and international partners [14]–[44], and thus data were collected and analyzed using quantitative methods. The accuracy of each item of data was scrutinized. Outliers and the normality of distributions were checked using frequent distributions and the Kolmogorov–Smirnov test. Descriptive and inferential statistics (e.g., percentage frequency distributions, means, and standard deviations) were implemented to report the outcomes. A Chi-square (*χ^2^*) analysis was applied to determine significant differences between trends in different categories such as sex, age, experience, and work status. All statistical analyses were performed using the IBM SPSS Statistics 26.0 software (IBM Corp., Armonk, NY, USA).

### 2.6. Ethical approval

The ethics, methods, and study protocol were approved (#05242023) by the ACSM's Work Group overseeing the survey according to the 2013 Declaration of Helsinki. The participants provided informed consent, confidentiality was assured, and all respondents did not include their names in the filled questionnaire. Data collection was made by researchers with no access to any of the participants. All procedures that involved the research study participants were approved by the ACSM.

## 3. Results

The present cross-sectional national survey gathered data from 322 respondents, representing a return rate of 11.6%. According to the demographic characteristics (Table 1), respondents from all Emirates (50.5% males) with diverse backgrounds, occupations, and experience levels participated in the study. In particular, 24.2% of respondents had over 20 years of professional experience in the industry and 5.3% had 10–20 years of experience, showing that almost one in three respondents were very experienced professionals. Moreover, 25% of the participants currently work as practitioners under several occupations, mostly as full-time (15%) and part-time personal trainers (5%), while 30% of respondents did not hold an academic credential in exercise science or a related field. Full-time work and commercial fitness centers were stated by 41.6% and 27%, respectively. Lastly, 42% of respondents were not certified through any international or domestic fitness certification agencies, and 30% of participants were undergraduate students. All H&F trends included in the survey were ranked from highest (most popular trend) to lowest (least popular trend) mean score, which are illustrated in Table 2. A comparison of the top 20 H&F trends among the UAE and other countries and regions is shown in Table 3. Moreover, trends were categorized into the following six groups, as reported elsewhere [43],[44]: trends related to i) fitness professionals; ii) fitness activities; iii) training modalities; iv) programs oriented to specific populations; v) technology; and iv) health. Table 4 presents a grouped approach of the comparative analysis of the top 20 H&F trends in the UAE and the world. Lastly, Table 5 provides a grouped comparative analysis of the top 10 fitness trends in the UAE based on age, experience, and career choice.

**Table 1.** Demographics of the survey respondents in the UAE.

Project		***N***	**%**
*Gender*			
	Female	155	48.3
	Male	162	50.5
	Prefer not to say	4	1.3
	No binary/Gender queer	1	0.3
*Age (years)*			
	18–21	98	30.5
	22–34	122	38.0
	35–44	78	24.3
	45–54	18	5.61
	55–64	3	0.9
	>65	2	0.6
*Region (Emirates)*			
	Dubai	129	40.1
	Abu Dhabi	136	42.2
	Sharjah	48	14.9
	Umm Al-Quwain	1	0.3
	Ajman	3	0.9
	Fujeirah	5	1.6
	Ras Al Khaimah	0	0
*Education*			
	Some high school	14	4.3
	High school diploma	82	25.5
	Bachelor's degree	159	49.4
	Master's degree	54	16.8
	Doctoral degree	13	4.0
*Certification (s)*			
	Focus Awards	14	4.3
	ARP/ACSM Certified Ringside Physician®	1	0.3
	ACSM Certified Group Exercise Instructor	13	4.0
	National Academy of Sports Medicine (NASM)	8	2.5
	ARP/ACSM Certified Ringside Physician	1	0.3
	National Academy of Sports Medicine (NASM)	8	2.5
	ACSM/NCPAD Certified Inclusive Fitness Trainer	5	1.6
	National Strength & Conditioning Association (NSCA)	6	1.9
	International Sports Sciences Association (ISSA)	11	3.4
	Exercise is Medicine	2	0.6
	Active IQ	27	8.4
	American Council on Exercise (ACE)	11	3.4
	Europe Active	6	1.9
	ACSM/NPAS Physical Activity in Public Health Specialist	3	0.9
	National Board for Health & Wellness Coaching (NBHWC)	2	0.6
	Fitness Mentors (FM)	4	1.2
	National Federation of Professional Trainers (NFPT)	3	0.9
	SCW Fitness Education (SCW)	1	0.3
	USA Weightlifting (USAW)	2	0.6
	PD Approval	5	1.6
	Yoga Alliance	3	0.9
	International Fitness Professionals Association (IFPA)	4	1.2
	International Sports Sciences Association (ISSA)	11	3.4
	ACSM Certified Personal Trainer®	25	7.7
	National Council on Strength & Fitness (NCSF)	1	0.3
	STOTT Pilates	3	0.9
	National Exercise and Sports Trainer Association (NESTA)	2	0.6
	National Council for Certified Personal Trainers (NCCPT)	1	0.3
	American Fitness Professionals & Associates (AFPA)	2	0.6
	Cooper Institute (CI)	2	0.6
	Not currently certified	136	42.1
	Other	101	31.3
*Primary profession*			
	Personal trainer (full-time)	48	14.9
	Personal trainer (part-time)	15	4.7
	Group exercise instructor	6	1.9
	Exercise physiologist	3	0.9
	Clinical exercise physiologist	2	0.6
	Program manager	4	1.2
	Health/Fitness director	5	1.6
	Strength coach	1	0.3
	Owner/Operator	11	3.4
	Health/Wellness coach	1	0.3
	Corporate health and wellness	4	1.2
	Athletics trainer	0	0.00
	Undergraduate student	96	29.8
	Graduate student	17	5.3
	Teacher	4	1.2
	Professor	12	3.7
	Medical professional (MD/DO, RN, Physical Therapist, Occupational Therapist)	19	5.9
	Registered dietician (RD, EDN, LD)	0	0
	Strength & Conditioning coach	15	4.7
	Other	60	18.6
*Experience (years)*			
	0–1	49	15.2
	1–3	43	13.4
	3–5	45	14.0
	5–7	40	12.4
	7–9	50	15.5
	10–20	17	5.4
	>20	78	24.2
	Not from health/Fitness industry	49	15.2
*Work status*			
	Full-time	134	41.6
	Part-time	36	11.2
	I am not exercise professional	146	45.3
	Other	6	1.9
*Member of UAE REPs and/or UAE Bodybuilding & Fitness Federation?*			
	Member of UAE REPs.	74	23.3
	Not a member of either organization	237	74.5
	Member of UAE Bodybuilding & Fitness Federation	7	2.2
*Work setting*			
	Private Practice/Own business	52	16.1
	Commercial fitness center	87	26.9
	Community-based facility or program (like a YMCA or JCC)	3	0.9
	Hospital/Medical center program/Department	18	5.6
	University recreation center or student wellness center	31	9.6
	Commercial recreation center	0	0.0
	Various locations/Freelancer	34	10.5
	Other	98	30.3

On average, exercise for weight loss was selected as the number one trend in the UAE's H&F sector. Specifically, six trends related to fitness activities (#3 Traditional Strength Training, #4 Bodyweight Training, #5 Boxing, Kickboxing and Mixed Martial Arts, #6 Youth Athletic Development, #7 Outdoor Activities, and #10 High-Intensity Interval Training), one related to the training modalities (#2 Personal Training), one related to programs oriented to specific populations (#1 exercise for weight loss), one related to health/recovery (#8 Massage), and one related to fitness professionals (#9 Employing Certified Exercise Professionals) were included in the top 10 most attractive H&F trends nationwide. No trends related to technology and health were ranked among the top 10 most popular trends in the UAE. Instead, several technology- and health-related options were ranked between #11 and #20. A grouped comparative analysis of the top 10 H&F trends based on age, experience, and career choice did not exhibit significant differences. However, females and males responded differently. In particular, females selected exercise for weight loss and personal training as the number one and two trends, respectively, while males selected the opposite. In terms of the third most popular trend, females selected bodyweight training, while males selected traditional strength training. No differences were observed for the first and second places based on the respondents' age and years of professional experience. However, major differences were found between practitioners and others who mostly worked in academia or were students.

Comparing the top 20 UAE's H&F for 2024 with the most recently published worldwide trends [29], 50% (10/20) of the top 20 trends included in the global list were not present in the UAE's list (reimbursement for qualified exercise professionals, functional fitness training, fitness programs for older adults, yoga, worksite health promotion, exercise for mental health, lifestyle medicine, exercise is medicine, data-driven training technology, and online personal training). On the other hand, 10 trends (bodyweight training, boxing, kickboxing and mixed martial arts, youth athletic development, outdoor fitness activities, high-intensity interval training, walking/running/cycling clubs, circuit training, health club and spa, exercise for children's health, and small group training) were included in the UAE's list, but not in the global trend list.

The chi-square test, including a contingency coefficient (CC), showed significant differences between males and females. Specifically, exercise for weight loss [*χ^2^* (27, *Ν* = 322) = 54.913, *p* = 0.001, CC = 0.382] and lifestyle medicine [*χ^2^* (27, *Ν* = 322) = 105.363, *p* = 0.032, CC = 0.497] were reported more frequently by females, while traditional strength training [*χ^2^* (27, *Ν* = 322) = 41.737, *p* = 0.035, CC = 0.339] was reported more frequently by males. Additionally, differences were found between young (18–34 years) and middle-aged (35–64 years) respondents. In particular, health clubs and spas [*χ^2^* (18, *Ν* = 322) = 41.207, *p* = 0.001, CC = 0.337] and yoga [*χ^2^* (18, *Ν* = 322) = 34.626, *p* = 0.011, CC = 0.312] were more popular among middle-aged respondents compared to young ones. Lastly, youth athletic development [*χ^2^* (9, *Ν* = 322) = 17.479, *p* = 0.042, CC = 0.227] was reported more frequently by non-practitioners (students) compared to practitioners (full-time and part-time).

**Table 2.** A comprehensive ranking of future H&F trends in the UAE.

#	**Trend**	**Score**
1	Exercise for weight loss	8.57 ± 1.91
2	Personal training	8.41 ± 2.06
3	Traditional strength training	8.16 ± 2.11
4	Bodyweight training	8.12 ± 2.08
5	Boxing, kickboxing, and mixed martial arts	8.09 ± 2.12
6	Youth athletic development	8.08 ± 2.14
7	Outdoor fitness activities	8.07 ± 2.13
8	Massage	8.05 ± 2.16
9	Employing certified exercise professionals	8.04 ± 2.14
10	High-intensity interval training	8.02 ± 2.04
11	Lifestyle medicine	7.98 ± 2.17
12	Wearable technology	7.98 ± 2.20
13	Health/Wellness coaching	7.97 ± 2.13
14	Walking/Running/Jogging/Cycling clubs	7.94 ± 2.27
15	Exercise is medicine	7.92 ± 2.26
16	Exercise for children's health	7.88 ± 2.23
17	Circuit training	7.85 ± 2.18
18	Health club and spa	7.82 ± 2.12
19	Small group training	7.82 ± 2.14
20	Post rehabilitation or disease/Condition maintenance classes	7.79 ± 2.30
21	Fitness programs for older adults	7.73 ± 2.34
22	Functional fitness training	7.72 ± 2.17
23	Pilates	7.71 ± 2.26
24	Stretch-based training	7.70 ± 2.21
25	Pre- and post-natal fitness	7.69 ± 2.17
26	Yoga	7.65 ± 2.45
27	Exercise for mental health	7.64 ± 2.42
28	Subscription based membership	7.57 ± 2.19
29	Balance and stabilization training	7.38 ± 2.37
30	Plyometric training	7.34 ± 2.40
31	Multidisciplinary work teams	7.33 ± 2.32
32	Myofascial release	7.26 ± 2.48
33	Low-cost and budget gyms	7.26 ± 2.40
34	Mobile exercise apps	7.25 ± 2.38
35	Data-driven training technology	7.25 ± 2.29
36	Influencer fitness	7.22 ± 2.56
37	Water immersion	7.21 ± 2.55
38	Home exercise gyms	7.20 ± 2.44
39	Dance-based workouts	7.17 ± 2.45
40	On-demand exercise classes	7.13 ± 2.35
41	Reimbursement for qualified exercise professionals	7.01 ± 2.53
42	Boutique fitness studios	6.93 ± 2.51
43	Aquatic exercise	6.93 ± 2.49
44	Worksite health promotion	6.86 ± 2.49
45	Online personal training	6.78 ± 2.60
46	Virtual reality exercise training	6.32 ± 2.76

Note: Scores are expressed as mean values ± standard deviation.

## 4. Discussion

A web-based survey targeting the top trends in the UAE's H&F sector took place for the first time nationwide, aiming to support gym operators, exercise professionals, educators, and suppliers to recognize the most promising trends linked to particular fitness services and programs. Additionally, an investigation of this kind may assist various industry stakeholders in improving customer retention, loyalty, and satisfaction by offering good practices and evidence-based training solutions nationwide.

### 4.1. What is most popular?

The outcomes of the first-ever survey of H&F trends in the UAE that replicated the ACSM's methodology [29] showed numerous similarities and differences with results observed in other recent national and regional cross-sectional studies that examined the top H&F trends [35]–[44] and regions [30]–[34]. On average, exercise for weight loss and personal training were ranked #1 and #2 in the UAE's survey, respectively, which is a finding that corroborates the main results reported in other international studies [29]. Moreover, these two trends were identified as the number one and two top options across most subgroup analyses based on sex, age, experience, and career choice. In particular, females ranked exercise for weight loss #1 compared to males (#2) in the UAE. However, exercise for weight loss seems to be an attractive space for entrepreneurs and exercise professionals working with plus-sized consumers in various fitness settings, given that this trend has been currently reported as one of the top H&F trends in Italy (#9), Spain (#6), Portugal (#3), Greece (#5), Cyprus (#14), Europe (#4), Brazil (#2), Chile (#2), Mexico (#1), Turkey (#1), Iran (#2), China (#1), the United States (#4), and worldwide (#4) [29],[39],[41],[43],[44]. Exercise for weight loss appears to be popular not only in the UAE, but also globally. This is important given that a very high percentage of the UAE population has an unhealthy weight and dietary choices, high sedentary behavior, and low physical activity levels. Strong evidence reveals that bodily movement for people living in larger bodies may be a high priority for these populations that represent the vast majority of the world's adult population nowadays [7],[8]. Taking that into account, exercise programs tailored to people with unhealthy weights seem to be a popular field for personal trainers and gym operators who seek to attract this emerging type of clientele, which is highly boosted by the increasing prevalence of overweight individuals and obesity worldwide [6]. Additionally, personal training was ranked #2, whereas small group training (#19) showed a lower popularity, supporting the implementation of client-centered and customized training sessions for consumers seeking to have exercise experiences in a private setting. This finding coincides with recently published data from other international surveys that identified the top H&F trends across the world (Table 3).

**Table 3.** Comparative analysis of the top 10 H&F trends between the UAE and other countries and regions.

**#**	**UAE**	**Australia** [29]	**Brazil** [29]	**Europe** [29]	**Mexico** [29]	**United States** [29]	**World** [29]
1	Exercise for weight loss	Exercise for mental health	Fitness programs for older adults	Personal training	Exercise for weight loss	Wearable technology	Wearable technology
2	Personal training	Fitness programs for older adults	Exercise for weight loss	High-intensity interval training	Personal training	Worksite health promotion	Worksite health promotion
3	Traditional strength training	Group training	Personal training	Small group training	Traditional strength training	Fitness programs for older adults	Fitness programs for older adults^2^
4	Bodyweight training	Functional fitness training	Functional fitness training	Exercise for weight loss	Training and feeding programs	Exercise for weight loss	Exercise for weight loss
5	Boxing, kickboxing, and mixed martial arts^1^	Exercise is medicine	Traditional strength training	Bodyweight training	Functional fitness training	Reimbursement for qualified exercise professionals	Reimbursement for qualified exercise professionals
6	Youth athletic development	Traditional strength training	Exercise for mental health	Functional fitness training	Youth athletic development	Employing certified exercise professionals	Employing certified exercise professionals
7	Outdoor fitness activities	Employing registered exercise professionals	Outcome measurements	Fitness programs for older adults	Healthy diet	Mobile exercise apps	Mobile exercise apps
8	Massage^1,2^	Pilates	Post-rehabilitation classes	Employing certified exercise professionals	Multidisciplinary work teams	Exercise for mental health	Exercise for mental health
9	Employing certified exercise professionals	Wearable technology	Walking/Running/Jogging/Cycling clubs	Exercise is medicine	Bodyweight training	Youth athletic development	Youth athletic development
10	High-intensity interval training	Inclusive exercise services	Outdoor fitness activities	Traditional strength training	Exercise for children's health	Personal training	Personal training

Note: ^1^appearance only in the UAE, ^2^region-specific trend.

**Table 4.** A grouped comparative analysis of the top 20 fitness trends in the UAE and the world.

**#**	**UAE**	**#**	**World** [29]
*Trends related to fitness professionals:*			
9	Employing certified exercise professionals	5	Reimbursement for qualified exercise professionals^3^
		6	Employing certified fitness professionals
*Trends related to fitness activities*			
3	Traditional strength training	9	Youth athletic development
4	Bodyweight training^1^	12	Outdoor activities
5	Boxing, kickboxing, and mixed martial arts^1^	14	Functional fitness training^3^
6	Youth athletic development	15	Yoga^2^
7	Outdoor fitness activities	17	Traditional strength training
10	High-intensity interval training	20	High-intensity interval training
14	Walking/Running/Cycling Clubs^1^		
17	Circuit training^1^		
18	Health club and spa^1^		
*Trends related to training modalities:*			
2	Personal training	10	Personal training
13	Health/Wellness coaching	13	Health/Wellness coaching
19	Small group training^1^		
*Trends related to programs oriented to specific populations:*			
1	Exercise for weight loss	3	Fitness programs for older adults^3^
16	Exercise for children's health^1^	4	Exercise for weight loss
*Trends related to technology:*			
12	Wearable technology	1	Wearable technology
		18	Data-driven training technology^3^
		19	Online personal training^3^
*Trends related to health:*			
8	Massage^1,2^	2	Worksite health promotion^3^
11	Lifestyle medicine	8	Exercise for mental health^3^
15	Exercise is medicine	11	Lifestyle medicine^3^
20	Post rehabilitation maintenance classes	16	Exercise is medicine^3^

Note: ^1^appearance only in the UAE, ^2^region-specific trend, ^3^appearance only in the worldwide survey.

**Table 5.** A grouped comparative analysis of the top 10 fitness trends in the UAE based on sex, age, experience, and work status.

**Sex**		**Age**		**Experience**		**Work Status**	
*Female (N = 162)*		*< 35 years (N = 220)*		*<10 years (N = 177)*		*Full and Part-time (N = 171)*	
Trend	Score	Trend	Score	Trend	Score	Trend	Score
ExWL	8.72 ± 1.87	ExWL	8.56 ± 1.89	PT	8.56 ± 1.96	PT	8.49 ± 2.05
PT	8.35 ± 2.18	PT	8.36 ± 2.09	ExWL	8.50 ± 1.89	ExWL	8.47 ± 1.90
BWT	8.22 ± 1.05	TST	8.26 ± 1.97	TST	8.27 ± 1.92	TST	8.27 ± 2.12
WT	8.20 ± 2.10	BKMMA	8.22 ± 2.07	BWT	8.26 ± 1.91	ECExP	8.26 ± 2.06
YAD	8.09 ± 2.16	YAD	8.21 ± 2.12	OFA	8.25 ± 2.01	Mas	8.05 ± 2.20
OFA	8.09 ± 2.14	OFA	8.17 ± 2.14	ECExP	8.22 ± 1.97	HIIT	8.04 ± 1.98
LsM	8.07 ± 2.07	BWT	8.11 ± 2.05	BKMMA	8.15 ± 2.08	BKMMA	8.01 ± 2.22
BKMMA	8.04 ± 2.14	Mas	8.08 ± 2.10	HWC	8.14 ± 1.95	CT	8.01 ± 2.14
Mas	8.01 ± 2.31	LsM	8.06 ± 2.07	ExM	8.13 ± 2.03	SGT	7.99 ± 2.14
TST	7.99 ± 2.10	ExM	8.05 ± 2.08	CT	8.12 ± 1.91	HWC	7.95 ± 2.14
*Male (N = 155)*		*>35 years (N = 101)*		*>10 years (N = 67)*		*Other (N = 151)*	
PT	8.52 ± 1.92	ExWL	8.60 ± 1.97	PT	8.69 ± 1.84	ExWL	8.70 ± 1.92
ExWL	8.48 ± 1.93	PT	8.52 ± 2.01	ExWL	8.63 ± 1.79	WT	8.36 ± 1.94
TST	8.39 ± 2.12	ECExP	8.29 ± 2.03	ECExP	8.39 ± 2.02	OFA	8.33 ± 1.91
ECExP	8.25 ± 2.09	HCS	8.13 ± 1.91	TST	8.28 ± 2.08	PT	8.33 ± 2.08
Mas	8.15 ± 1.96	BWT	8.13 ± 2.16	ExCH	8.22 ± 2.01	BWT	8.32 ± 1.92
BKMMA	8.14 ± 2.12	Yoga	8.08 ± 2.03	YAD	8.19 ± 2.14	YAD	8.30 ± 1.97
HIIT	8.12 ± 1.87	Mas	7.98 ± 2.31	BKMMA	8.10 ± 2.08	LsT	8.25 ± 1.90
YAD	8.10 ± 2.14	WT	7.96 ± 2.12	CT	8.09 ± 2.07	BKMMA	8.18 ± 1.99
OFA	8.08 ± 2.08	HIIT	7.95 ± 1.98	FFT	8.06 ± 1.99	WRJCC	8.14 ± 2.09
BWT	8.06 ± 2.13	TST	7.93 ± 2.38	OFA	8.06 ± 2.01	ExM	8.09 ± 2.12

Note: GExT, Group Exercise Training; PT, Personal Training; WT, Wearable Technology; YAD, Youth Athletic Development; OFA, Outdoor Fitness Activities; LsM, Lifestyle Medicine; BKMMA, Boxing, Kickboxing, and Mixed Martial Arts; Mas, Massage; TST, Traditional Strength Training; ExWL, Exercise for Weight Loss; ECExP, Employing Certified Exercise Professionals; HIIT, High-Intensity Interval Training; ExM, Exercise is Medicine ; BWT, Bodyweight Training; Core T, Core Training; HCS, Health Club and Spa; Yoga; HWC Health/Wellness Coaching; CT, Circuit Training; ExCH, Exercise for Children's Health; FFT Functional Fitness Training; SGT, Small Group Training; WRJCC, Walking/Running/Jogging/Cycling Clubs. Scores are expressed as mean values ± standard deviation.

Interestingly, 60% of the top 10 trends were related to various fitness activities in the UAE. More specifically, traditional strength training, bodyweight training, boxing, kickboxing, and mixed martial arts (MMA), youth athletic development, outdoor fitness activities, and high-intensity interval training were ranked as the most popular trends. The present findings are in line with those reported in various national and regional surveys. As for the most popular exercise settings, low-cost and budget gyms (#33) and boutique fitness studios (#42) were not present on the top trends list, not only in the UAE, but also globally (Table 3). Instead, health clubs and spas (#18) seem to be an emerging trend locally; however, it is not similarly popular internationally [29]. These observations cannot be analyzed here. Nevertheless, the low popularity of these particular types of fitness facilities in the UAE appears to be an important difference from other recently published international cross-sectional surveys. Importantly, fitness studios are on the rise among gym operators in mature fitness markets such as Europe and the USA [1],[2]. It is worth noticing that personal training studios have been ranked as the number one fitness setting among European exercise professionals, indicating the greatest potential for growth, innovation, and professional development [45]. This particular fitness facility type appears to be a promising business space, not only in developed countries but also in emerging markets, though not yet in the UAE [1],[11]. Instead, multi-purpose gyms currently demonstrate a higher popularity compared to boutique fitness studios in the UAE, and this is a major difference between the present study and others conducted in other countries and regions [29],[36],[37],[39],[43],[44]. Furthermore, bodyweight training (#4) and high-intensity interval training (#10) appear as popular fitness activities, showing that exercise programs combining those two trends in the same session may be a feasible, effective, and pleasant training option for practitioners and their clients in the UAE's H&F sector. This is an important observation supported by evidence since such a non-traditional, multi-component exercise approach has been recently highlighted as an evidence-based solution for health, fitness, and wellness among general and special populations [46]–[48].

### 4.2. Exercise professionals: Are they the gatekeepers to the H&F sector?

According to the main findings in the present study, fitness professionals-related trends, such as employing certified fitness professionals (#9), appear popular in the UAE. Similarly, this particular trend has been reported as attractive in other international observational studies of this kind [29],[34], revealing important similarities between the UAE and other countries regarding the minimum qualifications fitness professionals should meet to be eligible to enter the H&F sector. It is worth noticing that 49.4% and 42.1% of responders held at least a bachelor's degree in exercise science or a related field and/or a fitness certification, respectively (Table 1). However, considering that the present web-based survey was widely sent to local universities to collect data from students and academics involved in physiotherapy, fitness, and/or sports, this observation can be explained by the participants' demographics in the present study. Thus, a larger and more diverse sample may help a future investigation in this research area that focuses on the top trends in the UAE's H&F sector. Importantly, qualified fitness professionals serving general and special populations are necessary in the fight against the inactivity and obesity epidemics, aiming to support public health nationwide [6],[10]. That being said, established policies concerning education, certification, and lifelong learning among practitioners may be necessary for the proper regulation of this field initiated by Government entities. Such a strategy may promote the regulation and licensure requirements among practitioners while locally elevating the profession, aiming to protect consumers seeking high-quality fitness services and programs nationwide [49]–[52]. Additionally, the presence of three health-related trends among the top 20 in the UAE indicates the rationale for well-equipped practitioners, aiming to efficiently serve the masses.

### 4.3. What is no longer attractive?

Another important finding from the UAE's survey that should be taken into account is the low ranking of trends related to technology and mind-body fitness. In particular, Pilates (#23) and yoga (#26) were lowly ranked in the present survey, indicating that mind-body fitness modalities are not yet established in the UAE. However, respondents in the present survey were primarily young people that do not favor these training modalities, and thus this outcome needs further investigation in the future. Similarly, mind-body fitness activities have not been popular trends in various countries and regions (Table 3), and this is an outcome that might need more investigation in the future. However, both pilates and yoga have been documented as injury-free, enjoyable, and effective exercise training solutions, inducing beneficial changes in physical fitness, cardiovascular disease risk factors, and mental health [53]–[55].

Importantly, technology-oriented trends were not reported as popular in the post-COVID-19 era nationwide, excluding wearable technology (#12), since mobile exercise apps (#34), data-driven training technology (#35), and on-demand exercise classes (#40) were not included in the top 20 in the UAE. It is worth mentioning that the UAE's H&F sector is experiencing growth and expansion in this particular area; however, its development is still in the initial phase when compared to certain other nations. Specifically, digital services and online programs seem less popular among industry stakeholders during the post-COVID-19 era in the UAE. On the other hand, the impactful role of technology in the evolution and growth of the physical activity, exercise, and wellness sector has been well documented, revealing that digital fitness may be the game changer within the new fitness landscape [56],[57]. This outcome is aligned with the findings observed in various international surveys that detected an attractiveness of technology-related trends in numerous countries [35]–[44] and regions [30]–[34] (Table 3); however, the worldwide survey indicated that digital fitness-oriented trends were popular among exercise professionals [29] (Table 4). The factors behind the low popularity of technology-related trends in the UAE's H&F sector warrant further examination. Potentially, digital fatigue may overwhelm practitioners and consumers due to digital dependence during the COVID-10 lockdowns [58], and thus such services might be less attractive within the H&F sector. However, the future of the H&F sector is linked to the broad use of technology in various exercise settings [56],[59], which also affects professional education, certifications, and the lifelong learning context, with an emphasis on occupational roles related to health and well-being, as previously reported [60].

### 4.4. International comparisons

When comparing the UAE with the worldwide results, there were 11 matching trends from the top 20. Exercise for weight loss and personal training were within the top 10 in both surveys; however, the remaining UAE trends were more traditional and rooted activities, such as resistance training, bodyweight training, circuit training, and high-intensity interval training. The UAE, especially Dubai, is known for its innovative approach, though when it comes to exercise, traditional modalities dominate the list of trends. As a country-specific and additional trend, massages reached a high 8th position. This validates the recent surge in the popularity of sports and exercise recovery strategies in the UAE. An instance can be observed where the fitness programs for older adults held the significant 5th position in the world; however, these were not in the top 20 for the UAE. This revalidates recent research outcomes that indicated that a substantial number of older adults were overweight or obese, suggesting a trend of increasing obesity with age [60]. In summary, the top five fitness trends in the world, including worksite health promotion, exercise for mental health, and fitness programs for older adults, are products of well-established systematic work, policies, and regulations of the H&F sector. Contrary to that, the UAE trends are more likely a result of an individuals' self-awareness and understanding of the importance of being physically active. This is evident among the student demographic, as recent research conducted with a person-centered approach demonstrated a positive correlation between elevated health behavior patterns and an engagement in physical activities and healthy dietary practices [61]. A strategic comprehensive approach and policy initiatives by the Government would contribute to the overall improvement of the H&F of the UAE residents. Lastly, the UAE has implemented numerous initiatives and programs that focused on the younger generation in recent years and resulted in the elevated ranking of youth athletic development at a notable 6th position. This aligns with the UAE's National Sports Strategy 2031, which aims to identify and develop talent in schools [62]. Moreover, there has been a notable increase in the popularity of MMA disciplines, as evidenced by the survey placing boxing, kickboxing, and MMA at the 5th spot.

### 4.5. The impact of COVID-19

During the COVID-19 lockdown, all five aspects of unhealthy behaviors, including poor eating habits, inactivity, overweight/obesity, low sleep quality, and smoking, were on the rise in the UAE [63]–[66]. It has been well-reported that the H&F sector has been tremendously influenced by the COVID-19 pandemic globally, and resulted in a strong relationship between digital services and fitness businesses during the three-year global health crisis [1]. However, the rapid transition from in-person to online fitness services aggressively took place; therefore, technology-oriented trends were popular and necessary for consumers who exclusively sought virtual fitness programs throughout the COVID-19 pandemic [1]. Since the UAE rebounded from the COVID-19 pandemic relatively fast, an increment in face-to-face services has been widely reported in the present study. This is reflected even in the fitness trends results, as most of the top 20 trends included live workouts, either with individuals or within a group. Additionally, mobile exercise apps, on-demand exercise classes, online personal training, and virtual reality exercise training achieved relatively low scores. This outcome indicates that H&F providers should focus on in-person services compared to digital fitness services through a virtual exercise setting that was attractive to all industry stakeholders and consumers during the COVID-19 pandemic [32]–[34]. It is worth mentioning that the intention to use fitness apps noticeably increased in 2020–2023 globally [67], indicating that gym facilities offered digital fitness services and online exercise programs to their customers due to the pandemic regulations. Furthermore, outdoor activities were popular during the COVID-19 pandemic, since gyms adopted several restrictions to offer in-person fitness services worldwide [29],[34]. Interestingly, this particular trend retained its attractiveness after that challenging period, given that it was ranked #7 in the UAE and was also included in the top 20 H&F trends in other international surveys (Table 4) [29]. In Turkey, outdoor activities (#7) were selected as one of the most popular options, indicating that such fitness activities were no longer attractive nationwide. Likewise, walking, running, and cycling were reported as popular trends (#14) in the UAE. This is important since COVID-19 promoted aerobic-based activities out of the conventional gym setting, which enhanced the rapid growth of the global recreational running community [68] characterized by an accessible, cost-effective, and pleasant fitness activity. Lastly, group fitness programs were substantially influenced by the COVID-19 pandemic, showing extremely low popularity due to demanding sanitation protocols and limitations, and thus showed a low attractiveness globally in 2020–2022 [29],[33],[34]. Interestingly, group training is currently still unpopular not only in the UAE but also in various other countries and regions (Table 3). This is an observation that may highlight the strong impact of COVID-19 on a customers' intention and exercise behavioral regulation due to the 3-year cessation from regular engagement in group fitness classes.

### 4.6. Strengths and limitations

The biggest benefits of reporting the top H&F trends in the UAE will be for fitness companies, commercial gyms, and freelance personal trainers, considering that they will be able to align their services, programs, and products with the current trends in the H&F sector. Additionally, influential stakeholders and policy-makers can make informed decisions by utilizing data from the report. It is worth mentioning that the replication of the ACSM's methodology, which was broadly implemented in relevant international cross-sectional studies, may be an additional strength of the UAE's survey, underlining a high degree of standardization that may limit potential biases commonly reported in such observational studies. Importantly, the survey included UAE residents from a diverse range of nationalities, from 42 different countries, and spanned across five Emirates, thus providing important summarized data for the UAE.

On the other hand, cross-sectional surveys of this kind have numerous limitations based on the nature of the research design and methodology. More specifically, causal effects cannot be determined easily, and differences in the cohort and potential report biases are present, as previously articulated [36],[37],[39],[43],[44]. Thus, a lack of randomization and potential coverage errors in the sampling process are common in such observational studies. Moreover, due to the summer season and vacation time, the data collection period was shortened as people tended to take extended leaves and holidays during this season. This limited timeframe reduced the opportunity for comprehensive data collection, which prompted reopening the survey to collect further data. Additionally, the number of distributed surveys was restricted due to the reduced availability of individuals who could participate. Furthermore, the response rate to the survey was typically low during the selected period, resulting in a decreased engagement and participation. Lastly, there was a challenge in organizing and involving H&F entities in the project due to the constrained time frame.

### 4.7. Implications for future research

Given that the present study was focused on the professionals' opinions, further research targeting the consumers' viewpoint may be a beneficial approach for the local H&F sector. Such a new direction may support both the industry stakeholders and the community to spread the word that regular engagement in fitness services should be an important agenda item for locals living in a country presenting with sedentarism and increasing cardiovascular disease risk factors, such as obesity, type 2 diabetes, hypertension, and dyslipidemia [10]. Considering that such observational studies positively influence the local physical activity, exercise, and wellness sector by highlighting popular services, programs, and products, it may be important to continue to investigate H&F trends in the coming years in the UAE by including all Emirates and a large sample size that encompasses different age groups (from children to older adults) and expatriates from various countries across the world. Notably, the UAE's fitness market is transforming and emerging, engaging mostly young people and showing that the local market is immature compared to the USA and Europe [1]. On the other hand, young populations demonstrated insufficient physical activity, a poor sleep quality, high screen times, and unhealthy dietary habits, resulting in mental health impairments [62],[68]. However, the UAE presents the highest penetration rate among various Middle Eastern and North African countries, showing an opportunity to grow while enhancing customer engagement, satisfaction, and loyalty in the local H&F sector.

## 5. Conclusions

For the first time, a cross-sectional survey was carried out that focused on the current status of the H&F trends in the UAE. The present outcomes may benefit local gym operators/managers, fitness professionals, students, tutors, and academics to stay in line with the current status of the H&F trends in the UAE. The study indicated that a more conventional approach was popular when it came to physical activity and exercise training nationwide. Such research findings may benefit fitness companies, commercial gyms, and practitioners, considering that they can align their offers with the top national fitness trends in the post-COVID-19 era. Additionally, influential stakeholders and policy-makers can make informed decisions based on data from the present report.

## Use of AI tools declaration

The authors declare they have not used Artificial Intelligence (AI) tools in the creation of this article.

## References

[1] International Health, Racquet and Sports Club Association (2023). The 2023 IHRSA global report.

[2] Andreasson J, Johansson T (2014). Τhe fitness revolution: Historical transformations in the global gym and fitness culture. Sport Sci Rev.

[3] García-Fernández J, Gálvez-Ruiz P (2021). The global private health & fitness business: A marketing perspective.

[4] International Health, Racquet and SportsClub Association (2019). The 2019 IHRSA health club consumer report.

[5] Guthold R, Stevens GA, Riley LM (2018). Worldwide trends in insufficient physical activity from 2001 to 2016: A pooled analysis of 358 population-based surveys with 1.9 million participants. Lancet Glob Health.

[6] NCD Risk Factor Collaboration (NCD-RisC) (2017). Worldwide trends in body-mass index, underweight, overweight, and obesity from 1975 to 2016: A pooled analysis of 2416 population-based measurement studies in 128.9 million children, adolescents, and adults. Lancet.

[7] Sallis R (2015). Exercise is medicine: A call to action for physicians to assess and prescribe exercise. Phys Sportsmed.

[8] Rhodes RE, Janssen I, Bredin SS (2017). Physical activity: Health impact, prevalence, correlates and interventions. Psychol Health.

[9] International Monetary Fund (2023). World economic outlook database.

[10] World Health Organization (2018). Noncommunicable diseases country profiles 2018.

[11] Narula A, Chowcharia N (2021). UAE fitness services market outlook to 2025F-Driven by increasing health concerns resulting in addition of number of health clubs and gyms in the country.

[12] Thompson WR (2006). Worldwide survey reveals fitness trends for 2007. ACSM's Health Fit J.

[13] Thompson WR (2007). Worldwide survey reveals fitness trends for 2008. ACSM's Health Fit J.

[14] Thompson WR (2008). Worldwide survey reveals fitness trends for 2009. ACSM's Health Fit J.

[15] Thompson WR (2009). Worldwide survey reveals fitness trends for 2010. ACSM's Health Fit J.

[16] Thompson WR (2010). Worldwide survey reveals fitness trends for 2011. ACSM's Health Fit J.

[17] Thompson WR (2011). Worldwide survey reveals fitness trends for 2012. ACSM's Health Fit J.

[18] Thompson WR (2012). Worldwide survey reveals fitness trends for 2013. ACSM's Health Fit J.

[19] Thompson WR (2013). Now trending: worldwide survey of fitness trends for 2014. ACSM's Health Fit J.

[20] Thompson WR (2014). Worldwide survey of fitness trends for 2015: What's driving the market. ACSM's Health Fit J.

[21] Thompson WR (2015). Worldwide survey of fitness trends for 2016: 10th Anniversary Edition. ACSM's Health Fit J.

[22] Thompson WR (2016). Worldwide survey of fitness trends for 2017. ACSM's Health Fit J.

[23] Thompson WR (2017). Worldwide survey of fitness trends for 2018: The CREP edition. ACSM's Health Fit J.

[24] Thompson WR (2018). Worldwide survey of fitness trends for 2019. ACSM's Health Fit J.

[25] Thompson WR (2019). Worldwide survey of fitness trends for 2020. ACSM's Health Fit J.

[26] Thompson WR (2021). Worldwide survey reveals fitness trends for 2021. ACSM's Health Fit J.

[27] Thompson WR (2022). Worldwide survey reveals fitness trends for 2022. ACSM's Health Fit J.

[28] Thompson WR (2023). Worldwide survey reveals fitness trends for 2023. ACSM's Health Fit J.

[29] Newsome AM, Reed R, Sansone J (2024). 2024 ACSM worldwide fitness trends: future directions of the health and fitness industry. ACSM's Health Fit J.

[30] Kercher VM (2018). International comparisons: ACSM's worldwide survey of fitness trends. ACSM's Health Fit J.

[31] Kercher V, Feito Y, Yates B (2019). Regional comparisons: The worldwide survey of fitness trends. ACSM's Health Fit J.

[32] Kercher VM, Kercher K, Bennion T (2021). Fitness trends from around the globe. ACSM's Health Fit J.

[33] Kercher VM, Kercher K, Bennion T (2022). 2022 Fitness trends from around the globe. ACSM's Health Fit J.

[34] Kercher VM, Kercher K, Levy P (2023). Fitness trends from around the globe. ACSM's Health Fit J.

[35] Amaral PC, Palma DD (2019). Brazil and Argentina survey of fitness trends for 2020. ACSM's Health Fit J.

[36] Batrakoulis A, Chatzinikolaou A, Jamurtas AZ (2020). National survey of fitness trends in Greece for 2021. Int J Hum Mov Sports Sci.

[37] Batrakoulis A (2023). National survey of fitness trends in Greece for 2023. Int J Hum Mov Sports Sci.

[38] Batrakoulis A (2019). European survey of fitness trends for 2020. ACSM's Health Fit J.

[39] Batrakoulis A, Veiga OL, Franco S (2023). Health and fitness trends in Southern Europe for 2023: A cross-sectional survey. AIMS Public Health.

[40] Chávez LF, Zavalza AR, Rodríguez LE (2020). National survey of fitness trends in Mexico for 2020. Retos.

[41] Li YM, Han J, Liu Y (2019). China survey of fitness trends for 2020. ACSM's Health Fit J.

[42] Valcarce-Torrente M, Arroyo-Nieto A, Veiga OL (2022). National survey of fitness trends in Colombia for 2022. Retos.

[43] Batrakoulis A, Fatolahi S, Dinizadeh F (2023). Health and fitness trends in Iran for 2024: A cross-sectional study. AIMS Publlc Health.

[44] Batrakoulis A, Keskin K, Fatolahi S (2024). H&F trends in the post-COVID-19 era in Turkey: A cross-sectional study. Ann Appl Sport Sci.

[45] Gronau N, Titze G (2018). Personal training in Europe.

[46] Batrakoulis A, Jamurtas AZ, Fatouros IG (2021). High-intensity interval training in metabolic diseases: Physiological adaptations. ACSM's Health Fit J.

[47] Batrakoulis A, Fatouros IG (2022). Psychological adaptations to high-intensity interval training in overweight and obese adults: A topical review. Sports.

[48] Batrakoulis A, Jamurtas AZ, Metsios GS (2022). Comparative efficacy of 5 exercise types on cardiometabolic health in overweight and obese adults: A systematic review and network meta-analysis of randomized controlled trials. Circ Cardiovasc Qual Outcomes.

[49] Middelkamp J, Wolfhagen P, Eemstra J (2020). Group fitness in Europe.

[50] De Lyon ATC, Neville RD, Armour KM (2017). The role of fitness professionals in public health: A review of the literature. Quest.

[51] Soan EJ, Street SJ, Brownie SM (2014). Exercise physiologists: Essential players in interdisciplinary teams for noncommunicable chronic disease management. J Multidiscip Healthc.

[52] Muth ND, Vargo K, Bryant CX (2015). The role of the fitness professional in the clinical setting. Curr Sports Med. Rep.

[53] Batrakoulis A (2022). Psychophysiological adaptations to pilates training in overweight and obese individuals: A topical review. Diseases.

[54] Batrakoulis A (2022). Psychophysiological adaptations to yoga practice in overweight and obese individuals: A topical review. Diseases.

[55] Batrakoulis A (2023). Role of mind-body fitness in obesity. Diseases.

[56] García-Fernández J, Gálvez-Ruiz P (2022). The digital transformation of the fitness sector: A global perspective.

[57] Štajer V, Milovanovic IM, Todorovic N (2022). Let's (Tik) talk about fitness trends. Front Public Health.

[58] Gregersen EM, Astrupgaard SL, Jespersen MH (2023). Digital dependence: Online fatigue and coping strategies during the COVID-19 lockdown. Media Cult Soc.

[59] Middelkamp J, Rutgers H (2016). Growing the fitness sector through innovation.

[60] Ibtihal F, Razzak B, Abdul H (2019). National accountability and response for noncommunicable diseases in the United Arab Emirates. IJNCD.

[61] Webster C., Mîndrila D, Murphy A (2024). Student profiles of physical activity, screen time, sleep quality and dietary habits and their association with mental health and school satisfaction: An exploratory study. Psychol Sch.

[62] General Authority of Sports (2023). The national sports strategy-2031.

[63] Radwan H, Al Kitbi M, Hasan H (2021). Indirect health effects of COVID-19: Unhealthy lifestyle behaviors during the lockdown in the United Arab Emirates. Int J Environ Res Public Health.

[64] Arumugam A, Murat D, Javed A (2023). Association of sociodemographic factors with physical activity and sleep quality in Arab and non-Arab individuals of both sexes during the COVID-19 pandemic. Healthcare.

[65] Alsamman RA, Shousha TM, Faris ME (2024). Association of sociodemographic, anthropometric, and sleep quality factors with accelerometer-measured sitting and physical activity times among Emirati working women during the COVID-19 pandemic: A cross-sectional study. Womens Health (Lond).

[66] Angosto S, García-Fernández J, Grimaldi-Puyana M (2023). A systematic review of intention to use fitness apps (2020–2023). Humanit Soc Sci Commun.

[67] Dalibalta S, Majdalawieh A, Yousef S (2021). Objectively quantified physical activity and sedentary behaviour in a young UAE population. BMJ Open Sport Exerc Med.

[68] Georgiou Y, Patsantaras N, Kamberidou I (2024). The running tribes: Typology of the long-distance running community of Greece. Eur J Phys Educ.

